# Loss of compatibility might explain resistance of the *Arabidopsis thaliana* accession Te-0 to *Golovinomyces cichoracearum*

**DOI:** 10.1186/1471-2229-12-143

**Published:** 2012-08-11

**Authors:** Georgina Fabro, María Elena Alvarez

**Affiliations:** 1 Centro de Investigaciones en Química Biológica de Córdoba CIQUIBIC, UNC-CONICET, Departamento de Química Biológica, Facultad de Ciencias Químicas, Universidad Nacional de Córdoba, Haya de la Torre y Medina Allende, Ciudad Universitaria, Córdoba, X5000HUA, Argentina

**Keywords:** *A. thaliana*, *G. cichoracearum*, Compatibility factors, Powdery mildews, Conidiophore maturation, Loss of susceptibility, Haustorial staining, Haustorial imaging

## Abstract

**Background:**

The establishment of compatibility between plants and pathogens requires compliance with various conditions, such as recognition of the right host, suppression of defence mechanisms, and maintenance of an environment allowing pathogen reproduction. To date, most of the plant factors required to sustain compatibility remain unknown, with the few best characterized being those interfering with defence responses. A suitable system to study host compatibility factors is the interaction between *Arabidopsis thaliana* and the powdery mildew (PM) *Golovinomyces cichoracearum.* As an obligate biotrophic pathogen, this fungus must establish compatibility in order to perpetuate. In turn, *A. thaliana* displays natural variation for susceptibility to this invader, with some accessions showing full susceptibility (Col-0), and others monogenic dominant resistance (Kas-1). Interestingly, Te-0, among other accessions, displays recessive partial resistance to this PM.

**Results:**

In this study, we characterized the interaction of *G. cichoracearum* with Te-0 plants to investigate the basis of this plant resistance. We found that Te-0´s incompatibility was not associated with hyper-activation of host inducible defences. Te-0 plants allowed germination of conidia and development of functional haustoria, but could not support the formation of mature conidiophores. Using a suppressive subtractive hybridization technique, we identified plant genes showing differential expression between resistant Te-0 and susceptible Col-0 plants at the fungal pre-conidiation stage.

**Conclusions:**

Te-0 resistance is likely caused by loss of host compatibility and not by stimulation of inducible defences. Conidiophores formation is the main constraint for completion of fungal life cycle in Te-0 plants. The system here described allowed the identification of genes proposed as markers for susceptibility to this PM.

## Background

Plant disease is a rare event in natural environments, as constant improvement of defence programs effectively controls most microbial pathogens. In addition to pre-formed barriers, plants typically exhibit two lines of inducible defence responses. First, a surveillance system detects pathogens by sensing microbe-associated molecular patterns (MAMPs) or host-self modified components to activate pattern triggered immunity (PTI) [1]. This broad-spectrum immune response activates a complex signaling cascade, including the early and transient accumulation of reactive oxygen species (ROS), the deposition of the beta-glucan callose at the plant cell wall and a massive transcriptional reprogramming [2].

Although PTI effectively restricts the majority of “non-adapted” pathogens, particular “adapted” microbes can inactivate this pathway establishing compatible interactions with the plant. This latter condition implies an intimate communication between host and invader, and requires the coordinated action of pathogen-derived effectors over their host molecule targets [3-5]. These host targets are elements of defence signaling cascades, metabolic pathways, or structural cell components, whose modification by effectors favors pathogen’s nutrition or growth and thus can be considered as "compatibility factors" [6].

A second line of defence participates in the control of adapted pathogens. This response involves the action of resistance proteins (R) which recognize effectors, or products of their activity, to induce effector triggered immunity (ETI) providing race-specific resistance. PTI and ETI share several components but only ETI leads to the collapse of invaded cells, generating a Hypersensitive Response (HR) [7]. In addition, broad-spectrum resistance against several species of the same genera of pathogens may also display HR features [8]. The components of these pathways have been deeply characterized over the past decades, including the plant hormones salicylic acid (SA) and jasmonic acid (JA), whose mutual balance modulates resistance to biotrophic and necrotrophic pathogens [9]. In contrast, little is known about the nature or function of host compatibility factors supporting disease development. Forward and reverse genetic approaches have revealed a few monogenic recessive loci whose loss of function reduced disease, therefore suggesting they encode compatibility components [10]. Conversely, interactomic approaches such as yeast-two-hybrid and co-immunoprecipitation assays, mostly identified defence-related elements as targets of biotrophic or hemi-biotrophic pathogen effectors [11-13].

The interaction of plants with powdery mildews (PMs) offers suitable conditions for the study of compatibility and identification of plant and pathogen factors supporting disease development. PMs are adapted obligate pathogens *par* excellence, entirely depending on living tissue to grow and reproduce [10]. Compatibility with these pathogens requires a delicate balance between the successful extraction of resources and maintenance of host viability [14,15]. In particular, three Arabidopsis PM diseases have been well characterized, involving the following PM species: *Golovinomyces cichoracearum* (formerly *Erysiphe cichoracearum*) [16], *Golovinomyces orontii* (formerly *Erysiphe orontii*) [17], and *Erysiphe cruciferarum*[18]. These pathosystems were used to clone genes involved in broad-spectrum disease resistance [8] and non-host resistance [19-21], as well as genes encoding compatibility factors [22-25]. Additionally, it has been recently described that another PM species, named *Oidium neolycopersici*, is able to complete its life cycle in Arabidopsis [26]. Interestingly, different *A. thaliana* accessions display variable levels of susceptibility to *G. cichoracearum*. Among these, Tenela (Te-0) shows no macroscopic signs of disease, with a moderate resistance being conferred by a recessively inherited locus named RPW3 (recognition of powdery mildew 3) raising the possibility that plant components necessary for pathogen development or propagation are missing or altered in Te-0 plants [16]. We here evaluated whether Te-0 resistance may result from defects in the establishment of compatibility. The defence and infection features observed in this interaction, indicated that Te-0 plants hold the formation of functional haustoria, but not the maturation of conidiophores. Thus, this experimental system would result suitable for characterization of compatibility conditions affecting the final stages of PM differentiation.

## Results

### PTI/ETI-like responses in Te-0 plants infected with *G. cichoracearum* UCSC1

To evaluate the basis of Te-0 resistance to *G. cichoracearum*, we monitored the activation of defence markers in this interaction. As controls, we used the fully susceptible Columbia (Col-0) plant, and the resistant Kashmir (Kas-1) plant. In the last accession, *RPW8*, an atypical *R* gene confers broad-spectrum resistance to several PM species by inducing SA-dependent defences, ROS accumulation and cell death in a reaction similar to the HR activated by classical R genes [8,16,27]. Three cytological markers of PTI/HR induction were initially analyzed, by monitoring accumulation of ROS with 3,3 diaminobenzidine (DAB) [28], deposition of callose at plant cell wall with aniline blue [29,30], and host cell death with lactophenol trypan blue [31,32].

**Figure 1:**
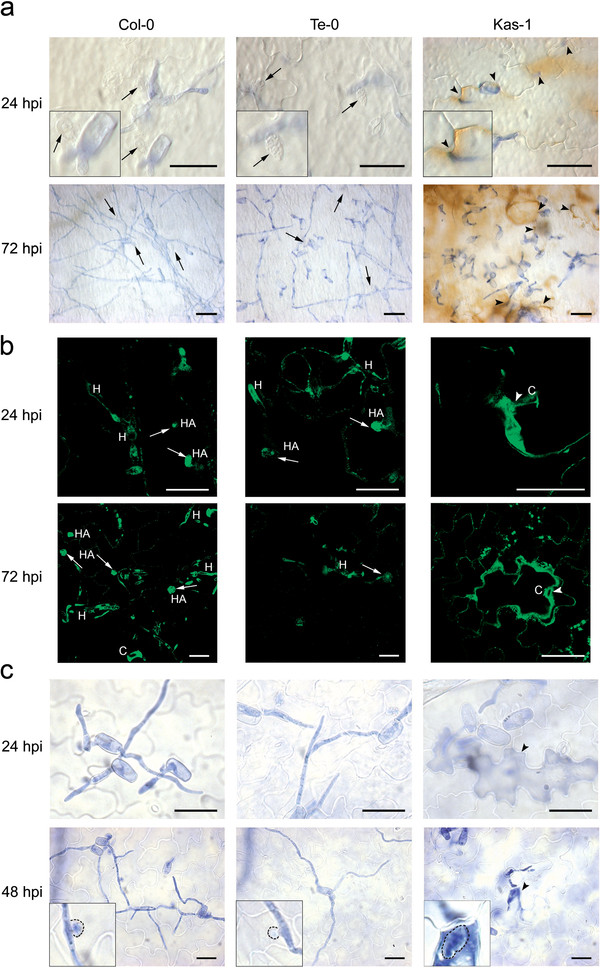
**PTI and ETI markers in *G. cichoracearum-*infected Col-0*, *Te-0, and Kas-1 plants.** (**a**) ROS accumulation revealed by the presence of brown-reddish precipitates in tissues treated with 3,3 diaminobenzidine (DAB). Fungal structures are stained with trypan blue. Haustoria (arrows) and attempted fungal penetration sites (arrowheads) are indicated. Bars: 30 μm. (**b**) Callose deposition evaluated by confocal microscopy of aniline blue stained leaves. Callose accumulates at the cell wall of Kas-1 epidermal cells contacting conidia (C; arrowheads). In Col-0 and Te-0 tissues, fluorescence is detected around the haustoria (HA; arrows), but not at the cell wall of penetrated cells. Fungal autofluorescence is also depicted. H: hyphae. Bars: 30 μm. (**c**) Plant cell death, evidenced by trypan blue staining, is only found in Kas-1 tissues (arrowhead). Haustorial-like structures are shown by dashed lines. Fungal structures are stained with trypan blue. Bars: 30 μm. Insets on (**a**) and (**c**) correspond to 4X enlargements of the shown pictures.

**Figure 2:**
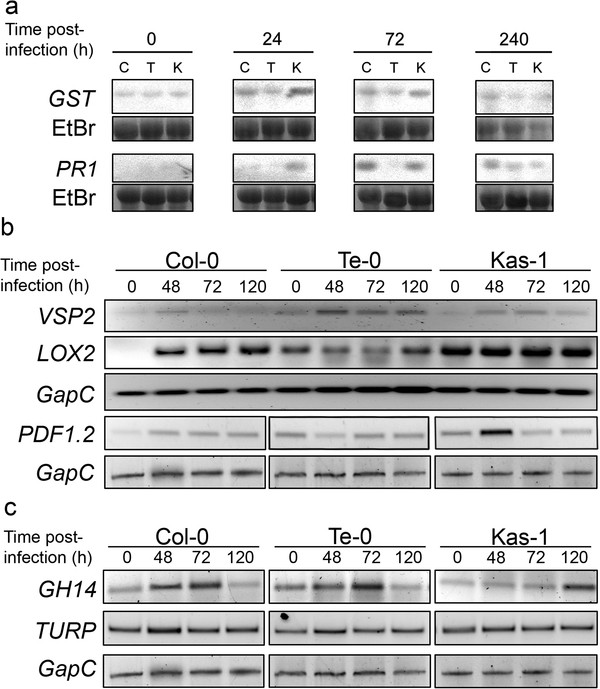
**Expression of defence genes on *G. cichoracearum-*infected tissues.** (**a**) Northern blots showing a time-course expression experiment for genes sensitive to ROS (*GST*) or SA accumulation (*PR1*) on Col-0 (C), Te-0 (T), and Kas-1 (K) plants. RNA was extracted from leaves at the indicated times post-infection with *G. cichoracearum.* (**b**) Semi-quantitative RT-PCR assays were used to evaluate the expression of the JA-sensitive genes *VSP2, LOX2* and *PDF1.2* on samples similar to those described on (**a**). *GapC* amplification was included as control for cDNA input. (**c**) Expression pattern of the *GH14* and *TURP* genes sensitive to the SA and JA pathways determined by semi-quantitative RT-PCR. These results were verified by qRT-PCR as shown in Additional file 2.

Accumulation of ROS was detected in infected Kas-1 plants (Figure 1a) where almost 50% of the interaction sites showed DAB staining at 24 hours post-inoculation (hpi). This response was stronger at 72 hpi reaching mesophyll cells close to conidia (Figure 1a, 72 hpi; Additional file 1a), resembling the response described for *RPW8*-mediated resistance [8]. In contrast, Te-0 and Col-0 tissues harboring haustoria did not accumulate ROS at 24 hpi (Figure 1a), with near 10% of the interaction sites showing DAB staining at 48 hpi (Additional file 1a). At 72 hpi, a faint DAB staining was detected in 20% (Col-0) or 26% (Te-0) of interaction sites, mainly in cells harboring old haustoria (Additional file 1a). Moreover, DAB precipitation was not observed in Te-0 epidermal/mesophyll cells supporting conidia with arrested germination (Figure 1a). In agreement with these results, *GST* (glutathione S-transferase) transcript accumulation, which usually accompanies redox alterations, was not detected in Te-0 infected tissues until 240 hpi (Figure 2a).

Callose deposits densely contouring epidermal cell walls were observed in Kas-1 infected tissues at 24 and 72 hpi, either at fungal contact sites or below non-germinated conidia (Figure 1b). Conversely, Te-0 and Col-0 infected epidermal cells, and adjacent mesophyll cells were devoid of callose deposits at the cell wall. In these two accessions fluorescence was detected around the haustoria (Figure 1b), probably as consequence of fungal auto-fluorescence and/or callose encasements becoming brighter with haustorial age [15,29,32,33].

As expected, dead cells were also observed in infected Kas-1 tissues. Near half of the epidermal cells contacting the pathogen showed trypan blue staining at 24 hpi (Additional file 1a). Later on, by 48 hpi, the few haustoria formed in these tissues were surrounded by plant stained cytoplasmic content (Figure 1c, inset). In addition, some mesophyll cells underlying the sites of interaction showed death signs in this plant (Additional file 1b). In contrast, cell death was almost absent in challenged Te-0 and Col-0 tissues until 72 hpi (Figure 1c; Additional file 1a).

In *A. thaliana*, susceptibility to biotrophic pathogens is modulated by the balance between the SA- and JA- dependent defence pathways [34-36]. We analyzed whether resistance of Te-0 plants to *G. cichoracearum* resulted from constitutive expression or hyper-activation of these pathways. For this purpose, we monitored the expression of the SA-sensitive gene marker *PR1* (pathogenesis-related gene 1), and the JA- inducible genes *LOX2* (lipoxygenase 2), *VSP2* (vegetative storage protein 2) and *PDF1.2* (beta-glucanase 1.2).

Uninfected tissues of Col-0, Te-0 and Kas-1 plants, showed no accumulation of *PR1* transcript. After infection, *PR1* activation was first detected in Kas-1 (24 hpi), then in Col-0 (72 hpi) and later on Te-0 plants (240 hpi) (Figure 2a). Concerning the JA marker genes, pathogen-mediated *VSP2* activation was weak and similar in all three plants (Figure 2b; Additional file 2). For *LOX2* and *PDF1.2*, these were up-regulated in uninfected Kas-1 and/or Te-0, with minor changes in expression after infection in Te-0 plants. Hence, the expression patterns of these gene markers suggested that the Te-0 plants showed neither strong basal stimulation nor fungal-induced activation of the SA and JA pathways. To search for signs of alterations in the equilibrium between the SA and JA pathways in Te-0, we examined the transcriptional activity of two genes sensitive to the interaction between these pathways in response to *G. cichoracearum*. The genes were the SA-dependent and JA-repressible glycoside hydrolase (*GH14*), and the JA-dependent and SA-repressible tumor related protein (*TURP*) [37]. We found that Col-0 and Te-0 plants expressed both genes with similar pattern during the course of infection (Figure 2c; Additional file 2).

In summary, our analysis suggested that resistance of Te-0 plants to *G. cichoracearum* is not accompanied by constitutive activation of PTI or exacerbation of ETI pathways.

### Limiting stages for development of *G. cichoracearum* on Te-0 plants

**Figure 3:**
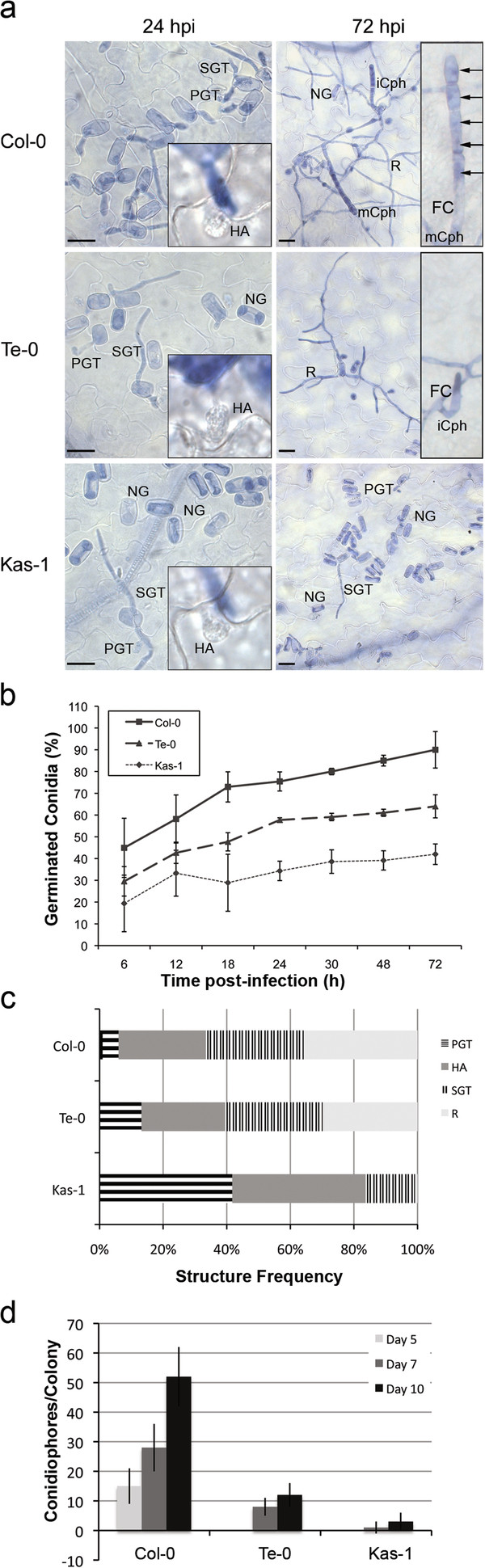
**The *G. cichoracearum* life cycle on Col-0, Te-0 and Kas-1 accessions.** (**a**) Fungal structures differentiated on leaf infected tissues. NG: non-germinated conidia; PGT: primary germ tube; SGT: secondary germ tube; R: ramified hyphae colony; FC: foot cell; iCph: immature conidiophore; mCph: mature conidiophore. Bars: 30 μm. Representative haustoria (HA), iCph and mCph found at the indicated times in similar samples are shown in the insets. (**b**) Conidial germination. Percentages of germinated conidia were obtained from the analysis of 10 inoculated leaves, and expressed as averaged weighed percentages ± coefficient of variance. Conidia showing PGT and subsequent differentiated structures were considered germinated. (**c**) Pattern of fungal differentiation at 48 hpi. The relative content of PGT, HA, SGT and R is indicated. Values are normalized to the number of germinated conidia. One representative experiment from three independent ones is shown. (**d**) Fungal asexual reproduction. The total content of conidiophores (mature plus immature) per colony is shown at 5, 7 and 10 days post-inoculation. Values resulted from the analysis of 100 colonies per accession (10–20 leaves from 5–10 different plants) and represented the average ± SD. Similar results were obtained in three independent experiments.

To analyze the progression of fungal disease in Te-0 tissues, we evaluated the abundance and morphology of pathogen-derived structures during an infection time-course assay. We performed the same analysis using susceptible Col-0 and resistant Kas-1 plants, as controls. For *G. cichoracearum*, conidial germination is followed by the formation of primary germ tubes (PGT), penetration of epidermal cells and generation of haustoria (HA) (Figure 3a) [16]. Then, secondary germ tubes (SGT) and ramified hyphae (R) contribute to form the fungal colonies, to finally differentiate the conidiophores (Cph), ending the fungal asexual reproductive cycle. Normally, a mature Cph (mCph) contains a five-conidia chain formed on a basal foot cell (FC). Occasionally, in some Arabidopsis genetic backgrounds displaying resistance to *G. cichoracearum*, this fungus only forms immature Cph (iCph) containing the FC and 1–2 conidia [34,38].

Leaves inoculated with conidia were sampled every 6 h during the initial 72 hpi, stained with trypan blue and analyzed by bright-field optical microscopy to quantify fungal structures (Figure 3; Additional file 3). Compared to Col-0 plants, conidial germination was severely reduced in Kas-1 but less affected in Te-0 plants (Figure 3a,b). These differences were maintained until 72 hpi, where 90.5 ± 8.4%, 64.2 ± 5.3% and 42.6 ± 4.7% of the inoculated conidia germinated in Col-0, Te-0 and Kas-1 plants, respectively (Figure 3b). After germination, at 48 hpi, the pattern of fungal structures developed on Te-0 tissues was very similar to the one found in Col-0, but substantially different to that observed on Kas-1 tissues (Figure 3c). At this time, comparable levels of R colonies were present in Te-0 and Col-0 plants (29 and 36% of germinated conidia, respectively), while this differentiation stage was never reached in Kas-1 plants, where fungal development was stopped at the PGT (41%) or HA (42%) stages (Figure 3c, Additional file 3c).

The fungal growth was delayed on Te-0 with respect to Col-0 plants in 6–12 h (Additional file 3a,b). Interestingly, despite the presence of R colonies in Te-0 plants (Figure 3c), the formation of Cph was severely reduced in these tissues (Figure 3d). By scoring the number of Cph (mCph and iCph) per colony, we found that Col-0 tissues harbored 15 ± 6 Cph/colony by 5 days post-inoculation (dpi) and a maximum of 52 ± 10 Cph/colony at 10 dpi, while Te-0 tissues showed no Cph at 5 dpi, and just 12 ± 4 Cph/colony at 10 dpi. This reduction in Cph content was maintained until late infection stages suggesting it was not merely due to delayed fungal growth on Te-0 plants. Although the fungal colonies formed in Te-0 were slightly smaller and less ramified than the ones developed in Col-0 (Figure 3a, 72 hpi), the number of haustoria present in colonies of comparable size was similar for both plants (12 ± 4 in Col-0, 10 ± 3 inTe-0, at 72 hpi). Strikingly, most Cph found in Te-0 showed an incomplete development as they only reached the stage of iCph (Figure 3a, 72 hpi).

In conclusion, conidia germinating in Te-0 tissues were able to differentiate HA and form R colonies, but never completed the formation of mCph, suggesting the latter process is a limiting step for *G. cichoracearum* asexual reproduction in this plant.

### Structural features of the fungal haustoria formed in Te-0 plants

**Figure 4:**
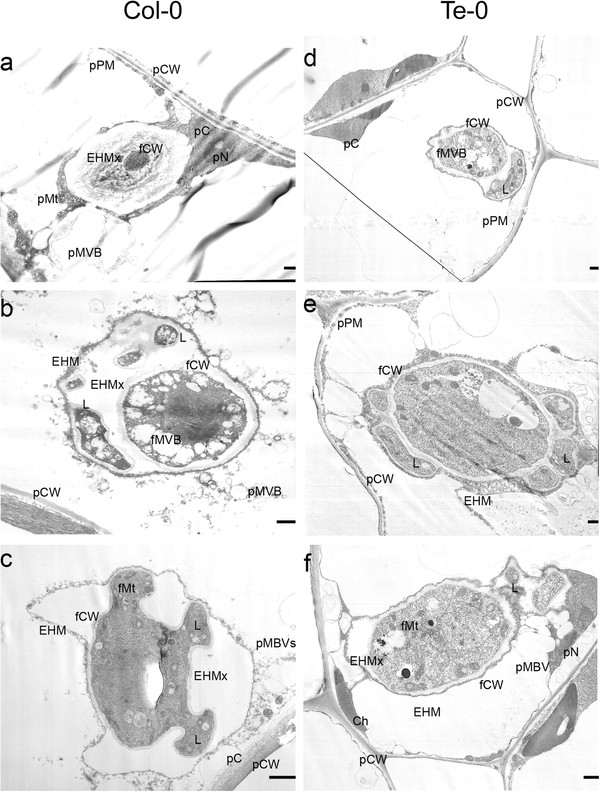
**Electron micrographs of *A. thaliana* epidermal cells harboring *G. cichoracearum* haustoria.** Ultra-structures of haustoria generated on susceptible Col-0 (**a**,**b**,**c**) and resistant Te-0 (**d**,**e**,**f**) plants. Images are representative of the most common observed phenotypes (n = 20 haustoria/plant). Pictures illustrate young (**a**,**d**) and mature (**b**,**c**,**e**,**f**) haustoria. pPM: plant plasma membrane; pCW: plant cell wall; pC: plant cytoplasm; pN: plant nucleus; pMVB: plant multi-vesicular bodies; pMt: plant mitochondria; fMt: fungal mitochondria; Ch: chloroplast; fCW: fungal cell wall; EHM: extra haustorial membrane; EHMx: extra haustorial matrix; fMVB: fungal multi-vesicular bodies; L: haustorial lobe. Bars: 1 μm.

Formation of haustoria is key for the establishment of compatible interactions with plants, since these structures deliver effectors into host cells and provide nutrients for fungal proliferation [39,40]. As Te-0 plants supported the generation of small fungal colonies with negligible amounts of mCph, we decided to evaluate the formation of haustoria in these tissues. Using electron microscopy, we compared the ultra-structural features of haustoria developed in Te-0 and Col-0 plants (Figure 4).

In these plants, the epidermal cells harboring haustoria showed no evidence of cell wall thickening or plasma membrane alterations (Figure 4a,d,c,f). Furthermore, no signs of organelle or cytoplasm disruption were found in these cells, which maintained intact nuclei located in the proximity of the haustorium (Figure 4a,f). Multi-lobbed haustoria (Figure 4b,c,e), and abundant mitochondria and multi-vesicular bodies congregated around or inside haustoria (Figure 4b,c,f) were present in both accessions. In addition, the electro-lucent appearance of the extrahaustorial matrix was similar in both plants (Figure 4c,e,f), and discontinuities or alterations in fungal cell wall and extra haustorial membrane were not observed in these tissues.

**Figure 5:**
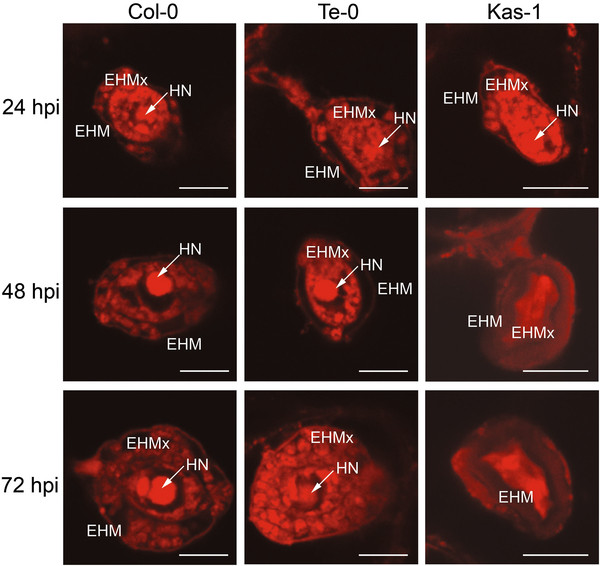
**Confocal microscopy imaging of *G. cichoracearum* haustoria.** Haustoria installed in plant epidermal cells are visualized by staining with sulpho-rodamine B (SRB) and 1-anilinonaphthalene-8-sulfonic acid (ANS). Z-stack compilations are shown for samples isolated at different times post infection (hpi). Arrows indicate the haustorial nuclei (HN) identified by DAPI staining (Additional file 4). EHM: extra haustorial membrane; EHMx: extra haustorial matrix. Bars: 10 μm.

To complement these studies, we compared the haustoria formed in Te-0 with those generated in Kas-1 plants. As no haustoria were present in ultra-microtome sections of Kas-1 infected tissues, we used confocal microscopy for this purpose. Here again, the haustoria developed on Col-0 plants were analyzed as control. Infected leaves were excised at 24, 48 and 72 hpi, to be fixed and then stained with sulpho-rodamine B (SRB) and 1-anilinonaphtalene-8-sulphonic acid (ANS) [41,42]. To our knowledge, this is the first evidence about the use of these compounds for visualization of fungal haustoria inside plant cells (Figure 5), as they have been traditionally used to stain proteins and lipids, respectively [41,42]. In parallel, DAPI staining was applied to identify the haustorial nucleus (Additional file 4).

At all examined stages, the haustoria generated in Te-0 were indistinguishable from those developed in Col-0, but clearly different from the ones present in Kas-1 plants, that actively counteract the fungal infection (Figure 5). Col-0 and Te-0 plants contained normal haustoria with single rounded nucleus, and vesicular-like compartments surrounded by a continuous thin structure likely corresponding to the EHM [15] (Figure 5). In contrast, 60-80% of haustoria formed on Kas-1 were abnormal at this time (Additional file 1a), presenting cytoplasmic disorganization and fainting of DAPI staining (Additional file 4), which suggested a degenerative process. In Col-0 and Te-0 these abnormalities were only observed at 72 hpi for a small subset of haustoria (18.00 ± 8.33 and 12.00 ± 9.33%, respectively, Additional file 1a). Using 3D reconstruction of images taken at 48 hpi, the maximum haustorial diameter was determined, revealing similar values for Te-0 and Col-0 samples (20.98 ± 2.49 and 21.52 ± 1.87 μm, respectively; *T*-test p < 0.05), and a significant reduction for haustorial diameter in Kas-1, respect to Col-0 samples (16.67 ± 3.96 μm, *T*-test p = 0.0034).

### Transport of L-arginine through haustoria established in Te-0 plants

The haustorium of obligate biotrophic fungi like powdery mildews and rusts participates in the absorption of sugars, amino acids, S, P, and other nutrients required for fungal growth [43-46]. To evaluate the functionality of the haustoria formed in Te-0, we analyzed their capacity to transport L-arginine C^14^ (ArgC^14^) from plant to fungal cells. In these experiments, leaf discs were excised at different times post-infection and floated for 5 h in ArgC^14^ solution. Then, the fungal material present on the disks surface was stripped using an adhesive tape and the amount of C^14^ in the tape was quantified. Sibling infected discs were sampled in parallel to determine the haustoria content per disc by trypan blue staining. In addition, discs from uninfected leaves were exposed to the same treatment to quantify and subtract the residual radioactivity transferred to the tape as result of the stripping process. Similar assays have been previously used for identical purpose [44,45].

We found that Kas-1 samples contained constant negligible levels of ArgC^14^ over the entire analyzed period (24–96 hpi; Additional file 5a). In contrast, Te-0-derived strips revealed an increase in the ArgC^14^ content during infection, with these values representing almost half of those corresponding to Col-0 samples until 72 hpi (Additional file 5a). Over this period, the differences observed may probably result from delayed fungal proliferation on Te-0 plants (Figure 3b,c, Additional file 3b). Considering this possibility, we normalized the levels of ArgC^14^ by the number of haustoria, and found no differences in the uptake of ArgC^14^ during the initial 72 hpi between mycelia installed in Te-0 and Col-0 plants (Additional file 5b). Interestingly, at 96 hpi, a net increase in radioactivity was detected in Col-0, but not in Te-0 samples. It was unknown whether this variation was also due to differences in fungal biomass. However, as maturation of Cph occurred at this time in the sensitive plant, the result might suggest that higher levels of nutrients are required under such condition. In agreement with this, such an increase was never reached, even at later time points, in Te-0 plants unable to hold mCph (these samples contained near 400 cpm of ArgC^14^ per stripped leaf disc at 120 hpi).

In conclusion, the results suggested that haustoria installed in Te-0 function in a very similar manner to those formed in Col-0 plants, at least with respect to the Arg uptake. Therefore, the conidiation deficiency observed in Te-0 would not result from severe failures in the absorption of nutrients.

### *A. thaliana* genes differentially expressed in Te-0 plants during the interaction with *G. cichoracearum*

To further characterize the interaction between Te-0 plants and *G. cichoracearum*, we identified genes having a differential expression in Col-0 and Te-0 tissues at late infection stages. For this purpose, cDNA libraries were generated from tissue samples isolated at 72 hpi, and used in suppressive subtractive hybridization assays (SSH) to confront Col-0 72 hpi cDNA samples (tester) against Te-0 72 hpi cDNAs samples (driver). The resulting non-subtracted cDNAs were cloned and sequenced, revealing 8 genes under-expressed in Te-0 compared to Col-0 samples. These differentially expressed genes encode for the following proteins: PR1, methallothionein 1a (MT1a), heat shock cognate protein 70.1 (HSP70.1), endoplasmin (END, HSP90.7), GRF7/GF14v 14-3-3 protein (GRF7), sucrose transporter 4 (STP4), chlorophyll a/b binding protein LHCB1.2, and an oxidoreductase. In principle, these genes could be considered markers of either ineffective defence activation or compatibility between *A. thaliana* and *G. cichoracearum*. We disregarded the study of defence-related genes encoding *PR1*, oxidoreductase, and chlorophyll a/b binding protein, whose role and/or behavior has been previously characterized for infections with this pathogen [36,37].

**Figure 6:**
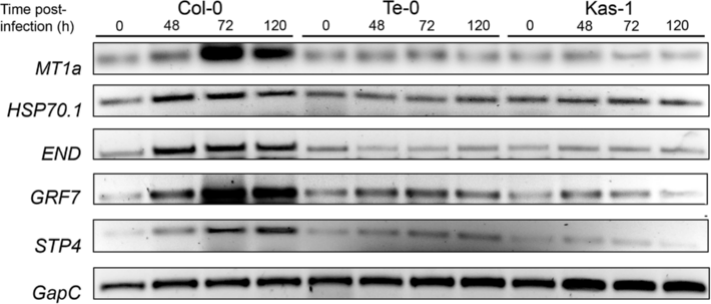
**Genes with differential expression in susceptible Col-0 and Te-0 resistant plants during the interaction with *G. cichoracearum*.** Gene expression is evaluated by semi-quantitative RT-PCR in uninfected and fungal infected leaves. *MT1a*: metallothionein 1a (*At1g07600*); *HSP70.1*: heat shock cognate protein 70.1 (*At5g02500*); *END*: endoplasmin (*At4g24190*); *GRF7*: 14-3-3 GF14v protein (*At3g02520*); and *STP4*: sucrose transport protein 4 (*At3g19930*). *GapC*: glyceraldehyde 3-phosphate dehydrogenase C subunit (*At3g04120*) used as control. These results were verified by qRT-PCR as shown in Additional file 6.

We selected the remaining five genes and monitored their expression in untreated and fungal-inoculated Col-0, Te-0 and Kas-1 leaves (Figure 6; Additional file 6). All genes became up-regulated in the Col-0 tissues sustaining fungal growth, with maximum expression at 48–72 hpi, and activation until 120 hpi. Consistent with the SSH assay, none of these genes were induced in Te-0 infected tissues, even at 120 hpi when the fungal biomass resembled that found in Col-0 tissues at 48 hpi (Additional file 5c). Interestingly, in Kas-1 plants the genes remained unaltered (*MT1a*, *HSP70.1, END)* or were down-regulated (*STP4, GRF7*) by infection, indicating their activation was not associated with *R*-gene mediated resistance but rather with compatibility with *G. cichoracearum*.

## Discussion

PMs establish long term relationships with their host forcing the maintenance of compatibility conditions [10]. The large and diverse collections of effectors encoded in the PM genomes [47] might indicate redundant functions of these compounds on their target molecules in the plant. This may explain why gene-for-gene resistance has negligible effect on the control of PM diseases in the field, and why plant resistance is overcome without apparent loss of fitness by the pathogen [48]. The identification of such effectors and their targets will be key to understand PM diseases, and design new alternatives to generate long-lasting passive resistance. To move in this direction, new experimental systems allowing the evaluation of compatibility as well as resistance by loss of susceptibility are required. In relation to the later condition, we have characterized the interaction between *A. thaliana* Te-0 plants and *G. cichoracearum*, where the host offers compatibility conditions during the initial, but not later stages of infection. In this plant, conidial germination is partially reduced and fungal growth is slightly delayed. Both features have been described in resistant interactions involving PTI or ETI/HR activation [8,20,30,34]. However, Te-0 infected tissues showed no signs of active defences (ROS accumulation, callose deposition, or cell death), in a response clearly distinguishable from that of infected Kas-1 tissues. Therefore, Te-0 resistance seems to be different from *RPW8*-mediated immunity, which confers broad-spectrum protection to powdery mildews in many different accessions of *A. thaliana* by activation of HR-like responses [8,27,49-51]. The SA-dependent pathway mediating resistance to biotrophic pathogens was neither induced under basal conditions, nor over-stimulated in Te-0 fungal-infected tissues. In addition, the JA signaling cascade did not seem to increase resistance against *G. cichoracearum* in Te-0 plants. The *LOX2* and *PDF1.2* gene markers had mild basal expression without activation after infection, whereas *VSP2* showed no basal expression and weak induction by pathogen, suggesting that resistance to the fungus was not due to major enhancement of JA-dependent defences. Curiously, the effect of the JA pathway on the interaction of *A. thaliana* with PMs is still unclear. The JA levels transiently increase in *A. thaliana* tissues infected with *G. cichoracearum*[37]. Resistance to PMs is enhanced in wild type plants treated with JA, as well as in *cev1* mutants that constitutively activate the JA pathway [52]. However, the *coi1-1* and *jar1-1* mutants, impaired in the JA-pathway, are not hyper-susceptible to PMs [53]. Therefore, in natural infection conditions, activation of the JA/ET pathway may not be sufficient to confer resistance to PMs [36].

Te-0 plants did not show signs of premature senescence, alterations in size, or developmental-induced callose accumulation (http://www.arabidopsis.org/ABRC and Additional file 7). Neither did these plants stimulate strong defence features upon fungal infection. These observations argue against the possibility that resistance of Te-0 plants might originate from mutations in genes encoding PMR2, PMR4, EDR1, EDR2 and PUX2, previously described as PM-susceptibility factors, causing hyper-activation of defence responses [22,25,34,38,54].

In *A. thaliana*, PMR5 and the pectate lyase PMR6 have been recognized as factors that potentiate susceptibility to PMs. PMR5 is involved in cell expansion whereas PMR6 functions in cell wall modeling [23,24], with both proteins being required for normal composition of plant cell wall pectin, and establishment of compatibility with PMs. Resistance of *pmr5*/*pmr6* mutants does not involve the SA or JA/ET pathways, resembling resistance of Te-0 plants. However, these mutants are stunted, show reduction in cell size, constitutive sporadic mesophyll cell death, and deposition of auto-fluorescent compounds along the veins. As none of these features were observed in healthy or *G. cichoracearum*-infected Te-0 tissues, it is likely that resistance does not result from deficient PMR5/6 function in these plants.

To date, the two best characterized limiting stages for development of PMs are penetration of epidermal cells [20,55] and development of haustoria [56,57]. Interestingly, *G. cichoracearum* was not prevented from reaching any of these stages in Te-0 plants. More than 50% of inoculated conidia germinated in Te-0 tissues further developing penetration hyphae and functional haustoria. In contrast, fungal asexual reproduction was the main limiting stage for proliferation of the pathogen in this plant. Limitations in this developmental stage have been previously described for PM diseases in *A. thaliana*[34,38]. In ascomycetes, coniditiation is regulated by several factors including inoculum density, light, temperature, humidity, and nutrient availability [58,59]. While significant progress has been made in elucidating this developmental program in model fungi (*Neurospora spp, Aspergillus spp*), its genetic basis and sensitivity to host clues remain elusive for obligate fungal biotrophs. In this sense, our results suggest that conidiation of *G. cichoracearum* in *A. thaliana* requires particular host conditions or signals which are present in Col-0, but not in Te-0 plants. Alternatively, Te-0 might offer limitations for optimal fungal development affecting for instance nutrient uptake, which negatively impact on Cph maturation, even though we found that the haustoria placed in these plants display normal assimilation of ArgC^14^.

Interestingly, Te-0 supports abundant mycelial development and moderate conidiation of the *G. cichoracearum* relative *Erysiphe cruciferarum*[18] and is susceptible to field isolates of the oomycete *Albugo spp.* (E Kemen, JD Jones, unpublished results). Thus, the following evidences support the possibility that Te-0 is altered in components necessary for compatibility with *G. cichoracearum*: i) genetic nature of plant resistance (recessive), ii) susceptibility of the plant to other biotrophic pathogens, including a close relative species; iii) absence of active defences (markers of PTI, ETI, SA and JA pathways) in this interaction.

To characterize gene expression changes occurring under compatibility conditions, we looked for transcriptional differences in Te-0- and Col-0 infected tissues at the pre-conidiation stage. By SSH, we identified 8 differentially expressed genes, including classical defence-related genes (*PR1*, oxidoreductase), and genes affecting photosynthesis (chlorophyll a/b binding protein), which have been characterized elsewhere [36,37]. The remaining 5 candidates (*MT1a, HSP70.1*, *END*, *GRF7* and *STP4*) showed increased expression in response to pathogen in susceptible Col-0 plants, but not in resistant Kas-1 plants, indicating they are sensitive to compatibility conditions, but not to ETI activation.

Previous studies reported activation of some of these genes in response to fungal infections. This has been observed for *STP4* during the interaction of *A. thaliana* with *G. cichoracearum*[60], and for *MT1a* and its rice homologue in response to other haustoria-forming fungi [61,62]. In turn, *HSP70.1* is induced by the obligate biotrophic oomycete *Hyaloperonospora arabidopsidis* (ex. *parasitica*) [63], whereas the *END-*[64], and *GRF7-*homologues in barley are up-regulated by PMs [65].

Depletion of genes encoding host compatibility factors is predicted to reduce pathogen growth. However, the functional redundancy of these genes might hinder the effect of null mutations on single genes. Conversely, an increased expression of one of these genes may generate tractable effects on the invader’s growth. Consistent with this prediction, *A. thaliana* plants over-expressing *HSP70.1* are hyper-susceptible to *H. arabidopsidis*[62], and rice plants activating *GRFs* show negative effects on defence-gene induction and cell death [66]. Furthermore, activation of *MT1a* leads to increased susceptibility to bacterial pathogens and reduced ROS accumulation in *Casuarina glauca*[67]. Curiously, plants over-expressing *END* are more sensitive to salt and drought stresses, although their responsiveness to pathogens has not been evaluated [68]. Therefore, taking these results together, the genes selected by our studies appear to be involved in responses to stress, with some of them acting in cell viability pathways.

## Conclusions

This study provides a detailed characterization of the interaction between the obligate biotrophic pathogen *G. cichoracearum* and the natural variant of the *A. thaliana* species, Te-0. We found that the limiting stage for fungal development *in planta* is the formation of condiophores. This limitation did not correlate with activation of cellular or molecular markers of PTI and ETI/HR. Considering the genetic bases reported for resistance of Te-0 plants (recessive and monogenic) [16], we propose this phenotype may result from the inability of the plant to maintain compatibility conditions at later stages of infection. As Te-0 did not show developmental or reproductive defects described in mutants lacking compatibility factors (*pmr2/4/5/6*), its resistance would derive from alterations in novel compatibility factors. A preliminary search for such factors allowed identification of candidate compatibility gene markers that must be characterized in the future. Furthermore, this work raises novel questions related to the control of *G. cichoracearum* asexual reproduction *in planta*, the existence of putative host molecular clues supporting this stage, and the contribution of the genes here identified to the establishment of compatibility with PMs.

## Methods

### Plant and fungal material

*A. thaliana* seeds were obtained from ABRC (http://www.arabidopsis.org/ABRC). Surface sterilized, stratified seeds were germinated on GM plates for 10 days, and transferred to soil. Plants were grown at 80% humidity, 21-23°C, 8/16 h light/dark cycles, 100 μE m^-2^ sec^-1^ irradiance and used at the age of 5–7 weeks. *G. cichoracearum* UCSC1 was obtained from Dr. Shauna Somerville [16] and propagated in Col-0 and squash (*Curcubita maxima* var. Kuta; Park Seed, Greenwood, SC, USA). Pathogen inoculation on leaf tissues was performed as previously described [37].

### Histochemical assays

To evaluate plant cell death, pathogen-infected leaves were treated with lactophenol trypan blue staining mix [31]. Samples mounted on glycerol 10% were observed with an Axioplan 135 microscope (Zeiss, Germany) to obtain images with an Axiovision camera system. Accumulation of ROS was detected by DAB (3,3 diaminobenzidine) staining [28]. Briefly, petioles were sectioned under water to avoid air bubbles in the vascular system. Leaves were dipped in 200 μl of DAB (1 mg/ml water, pH = 3.8) for 8 h, bleached in 96% ethanol for 24 hr, re-hydrated, mounted in glycerol 50% and observed as described for cell death. Fungal infective structures were detected and quantified by co-staining with trypan blue [31,37]. For callose detection, leaves were de-stained in ethanol 96%, re-hydrated in water and dipped in aniline blue solution (0.01%p/v in phosphate buffer 150 mM, pH = 9.5) for 1 hr. Leaves were mounted in 50% glycerol, and observed by confocal laser microscopy (Carl Zeiss Laser Scanning system LSM5 Pascal mounted in an inverted microscope Axiovert 200, Zeiss, Germany) using a 488 nm Argon laser and a c-apochromat 40X objective.

### Confocal microscopy

A solution of sulpho-rodamine B (SRB) plus 1-anilinonaphthalene-8-sulfonic acid (ANS) was used to stain fungal haustoria in infected plant tissues. Briefly, excised infected leaves were fixed for 1 h in 2,5% glutaraldehyde, 2,5% paraformaldehyde, and incubated for 20 min in solution containing 2 mg SRB (Molecular Probes, Eugene, OR, USA) and 1 mg ANS (Molecular Probes, Eugene, OR, USA) dissolved in 45 mL of methanol plus 45 mL MilliQ water and 10 mL of acetic acid. Leaves were rinsed for 10 min in PBS and mounted in 50% glycerol. Fluorescent structures were observed by confocal microscopy using the 543 nm laser and the c-apochromat 100X oil DIC objective.

### Electron microscopy

Infected leaves were sampled and fixed for 1 h in 2% paraformaldehyde, 2,5% gluteraldehyde. Fixed samples were included in Spurr resin [69], stained with osmium tetroxide, sectioned and further processed by the laboratory of electronic microscopy of the IFFIVE (INTA, Cordoba, Argentina).

### Fungal amino-acid uptake

Based on previous protocols [44,45], plants inoculated with conidia (ca. 500 mm^2^^-1^), were used to excise leaf discs (2 discs and 20 leaves per gentoype) at different time points. Two sets of discs (20 discs each) were floated either in water or solution of C^14^ L-Arginine 100 μM (Arg C^14^) for 5 h on a humid chamber, avoiding contact of mycelia with the solution. Leaf discs were deposited on tissue paper and the mycelium was stripped from the adaxial surface using an adhesive tape (Magic Tape, 3 M). Arg C^14^ content was determined on the tapes with scintillation counter. Using an identical procedure, discs taken from uninfected plants were used to subtract the radioactivity transferred to the tapes by mechanical damage of tissues. The number of haustoria on samples was determined by using trypan blue staining of the water-floated discs. A similar set of samples was used to estimate fungal biomass. Briefly, 15 Col-0 and Te-0 leaves (3 leaves/plant, 5 plants) were excised and stained with trypan blue at 48, 72 and 120 hpi to determine the leaf area (length x width) covered by fungal colonies using bright field microscopy images (Additional file 5c).

### Gene expression

Total RNA was isolated from healthy and *G. cichoracearum-*infected *A. thaliana* leaves [70] and used for Northern blot [37]. Ten micrograms of RNA per sample were blotted and hybridized with radioactive probes generated by random priming using dATP P^32^ (NEB) of the cDNAs obtained from the ABRC (*PR1*: U11550, *GST*: U13302). Total RNA extracted from uninfected (T0) or fungal infected (T72) Col-0 and Te-0 leaves were used for Suppresive Subtractive Hybridization (SSH) assays (Clontech’s PCR Select cDNA Subtraction Kit), following the manufacturer´s instructions. Subtracted cDNAs were cloned and sequenced.

One milligram of *A. thaliana* total RNA was treated with DNAse I (Invitrogene) for 1 h at 37°C to then be heated at 75°C. Random hexamer primers and MMLV reverse transcriptase (Invitrogen) were added to synthesize cDNA in 20 μl. One μl was used to perform either semi-quantitative (sq) or real time/quantitative (q) PCR (final volume: 25 or 14 μl, respectively).

sqRT-PCR was performed at least two times on biological replicate samples with similar results. Cycle number was tested for each gene to identify the linear phase of the PCR related to the initial amount of cDNA input. Primer sets and cycle number for each gene are stated in Additional file 8.

qPCR assays including the cDNA samples evaluated in Figure 2 and 6, were performed by triplicate using the Biodynamics mix B124-100 containing hot start Taq polymerase and SYBR green fluorophore, and the following protocol in a Rotor GeneQ thermocycler (Qiagen): 95°C for 10 min, then 40 cycles of 95°C for 15 s, 60°C for 15 s, 72°C for 30s, followed by a melting curve (60–95°C). Gene expression was calculated using the ΔΔCt method using *UQB5* as housekeeping control. Correction by the primer’s efficiency was performed. Efficiency was determined by developing control reactions with 1/10 dilutions of cDNA from Col-0 T0.

## Authors’ contributions

MEA designed and supervised the study, GF performed the experiments. GF and MEA analyzed the results and wrote the manuscript. All authors have read and approved the manuscript.

## Author’s information

GF and MEA are members of the Researcher Career of CONICET.

## Supplementary Material

Additional file 1 **a) Time-course quantification of cellular defence responses to *G. cichoracearum* in Te-0, Col-0 and Kas-1 plants.** 1- DAB stained cells in contact with *G. cichoracearum* structures*.* (*) No significant differences between these values in *T*-test at p < 0.05. 2- Trypan blue stained plant cells observed by bright field microscopy. 3- Haustoria stained with SRB-ANS were observed by confocal microscopy at the stated times post-inoculation. Abnormal haustoria are shown in Figure 5 (Kas-1, 48 hpi). In all these experiments 150 fungal interaction sites (1,2) or 150 haustoria (3) were evaluated for each genotype at the indicated times. Three independent experiments were performed. Values represent weighted average percentage ± coeficent of variance of the independent experiments. b) Mesophyll cell death evaluated on Kas-1 leaves infected with *G. cichoracearum* at 48 hpi by trypan blue staining. Left: focus on epidermal cells interacting with the pathogen. Right: underlying mesophyll dead cells. Click here for file

Additional file 2 **Expression of defence genes on *G. cichoracearum*-infected tissues assessed by qRT-PCR.** Values were obtained applying the ΔΔCt method using UBQ5 as housekeeping gene control. Bars represent average ± SD from three replicates. Click here for file

Additional file 3 **Time course development of *G. cichoracearum* infective structures on Col-0, Te-0 and Kas-1 accessions.** The abundance of different fungal structures is evaluated at the indicated time points. PGT: primary germ tube, HA: haustorium, SGT: secondary germ tube, R: ramified hyphae colony. Tissues were inoculated with the same conidial suspension. Values are referred as percentages of inoculated conidia. Click here for file

Additional file 4 **DAPI staining of the *G. cichoracearum* haustoria developed in Col-0, Te-0 and Kas-1 plants.** Haustoria were co-stained with SRB, ANS and DAPI. Z-stacks are shown for representative haustoria at 72 hpi. HN: haustorial nucleus. Bars: 10 μm. Click here for file

Additional file 5 **Uptake of ArgC^14^ by *G. cichoracearum* haustoria installed in Te-0 cells.** a) Uptake of ArgC^14^ by the fungal tissues differentiated on Col-0, Te-0 and Kas-1 leaves. The indicated values were obtained after subtracting residual radioactivity levels present on tapes stripped from uninfected leaves (see experimental procedures). b) ArgC^14^ levels detected in fungal tissue expressed as a ratio over the number of haustoria present on sibling leaf discs. c) Time-course estimation of the fungal biomass found on the surface of Col-0 and Te-0 leaves infected with *G. cichoracearum*. Fifteen leaves per plant were analyzed per time-point. Average percentage ± SD of leaf areas covered by fungal colonies is depicted. Click here for file

Additional file 6 **Genes with differential expression in susceptible Col-0 and Te-0 resistant plants during the interaction with *G. cichoracearum* evaluated by q-RT-PCR.** Fold changes in transcript expression levels were determined as described in Additional file 2. Bars represent average ± SD from three replicates, except for *STP4*, where q-PCR assay included two replicates. Click here for file

Additional file 7 **Uninfected Te-0 plants do not accumulate callose.** Untreated leaves from Te-0 plants (5-week-old, soil grown) were collected and stained with aniline blue to reveal callose by confocal microscopy using UV light. This assay was performed in parallel, under identical conditions to that shown in Figure 1b, where deposition of callose was detected in Kas-1 infected samples. Col-0 was included for comparison. Click here for file

Additional file 8 **Primers and conditions used for semi-quantitative and real time/quantitative RT-PCR assays.** (Ψ) Number of cycles used for sq-RT-PCR. (*) Different melting temperatures (Tm) were used in sq and q-RT-PCR. All q-RT-PCR were done with Tm = 60°C, except for *PDF1-2*. (^) *UBQ5* and *GapC* were used as housekeeping control genes in q-RT-PCR and sq-RT-PCR, respectively. Click here for file
