# Supplementary material for: Loss of compatibility might explain resistance of the Arabidopsis thaliana accession Te-0 to Golovinomyces cichoracearum
Source: BMC Plant Biol. 2012 Aug 11;12:143. doi: 10.1186/1471-2229-12-143 (PMC3546952; doi:10.1186/1471-2229-12-143)
Supplement: Additional file 1 — a) Time-course quantification of cellular defence responses to G. cichoracearum in Te-0, Col-0 and Kas-1 plants. 1- DAB stained cells in contact with G. cichoracearum structures. (*) No significant differences between these values in T-test at p < 0.05. 2- Trypan blue stained plant cells observed by bright field microscopy. 3- Haustoria stained with SRB-ANS were observed by confocal microscopy at the stated times post-inoculation. Abnormal haustoria are shown in Figure 5 (Kas-1, 48 hpi). In all these experiments 150 fungal interaction sites (1,2) or 150 haustoria (3) were evaluated for each genotype at the indicated times. Three independent experiments were performed. Values represent weighted average percentage ± coeficent of variance of the independent experiments. b) Mesophyll cell death evaluated on Kas-1 leaves infected with G. cichoracearum at 48 hpi by trypan blue staining. Left: focus on epidermal cells interacting with the pathogen. Right: underlying mesophyll dead cells. [file 1471-2229-12-143-S1.doc]

**Additional file 1.**

**a) Time-course quantification of cellular defence responses to *G. cichoracearum* in Te-0, Col-0 and Kas-1 plants.**

| **Accession** | **Time post- infection (h)** | **Host cells accumulating ROS^1^** | **Host cell death^2^** | **Abnormal haustoria^3^** |
| --- | --- | --- | --- | --- |
| Col-0 | 24 | 5.63 ± 1.82 | 3.36 ± 1.22 | 0 |
|  | 48 | 8.75 ± 2.35 | 6.67 ± 1.75 | 3.36 ± 6.00 |
|  | 72 | 19.65 ± 3.12 * | 6.25 ± 2.33 | 18.00 ± 8.33 |
| Te-0 | 24 | 5.20 ± 2.80 | 2.93 ± 1.25 | 0 |
|  | 48 | 10.50 ± 2.20 | 3.33 ± 2.72 | 4.33 ± 5.00 |
|  | 72 | 26.40 ± 4.70 * | 3.22 ± 2.86 | 12.00 ± 9.33 |
| Kas-1 | 24 | 54.20 ± 5.10 | 49.0 ± 4.0 | 34.00 ± 8.33 |
|  | 48 | 62.33 ± 8.30 | 69.1 ± 7.2 | 60.00 ± 13.33 |
|  | 72 | 70.70 ± 9.30 | 78.3 ± 9.2 | 83.33 ± 23.33 |

1- DAB stained cells in contact with *G. cichoracearum* structures*.* (*) No significant differences between these values in T-test at p< 0.05.

2- Trypan blue stained plant cells observed by bright field microscopy.

3- Haustoria stained with SRB-ANS were observed by confocal microscopy at the stated times post-inoculation. Abnormal haustoria are shown in Figure 5 (Kas-1, 48 hpi).

In all these experiments 150 fungal interaction sites (1,2) or 150 haustoria (3) were evaluated for each genotype at the indicated times. Three independent experiments were performed. Values represent weighted average percentage ± coeficent of variance of the independent experiments.

**b) Mesophyll cell death on Kas-1 leaves infected with *G. cichoracearum*.**


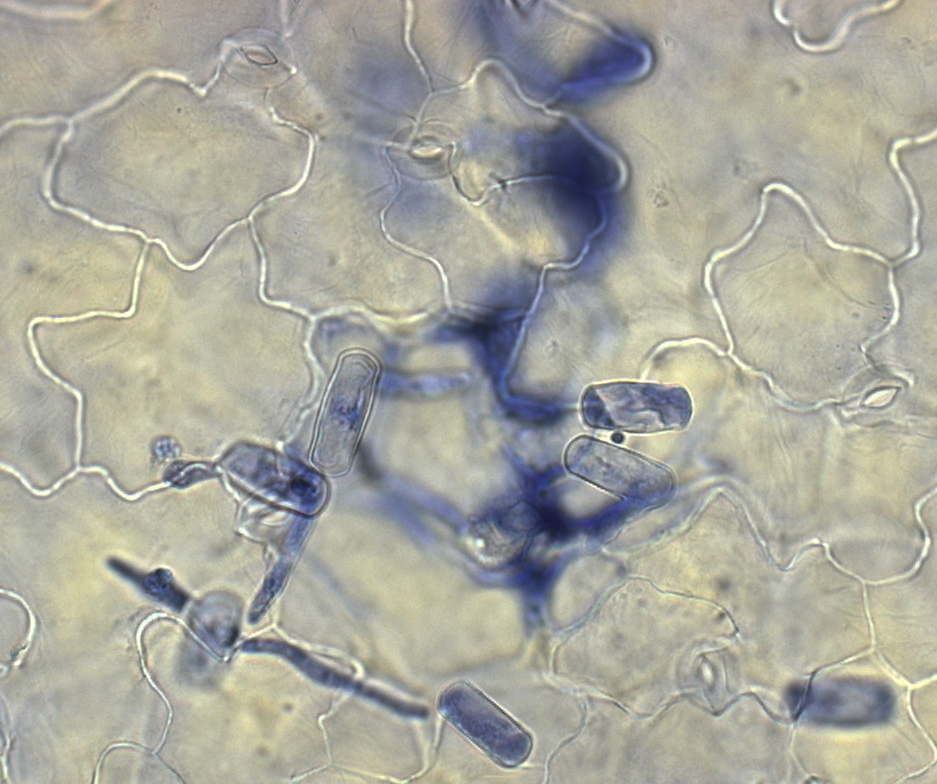

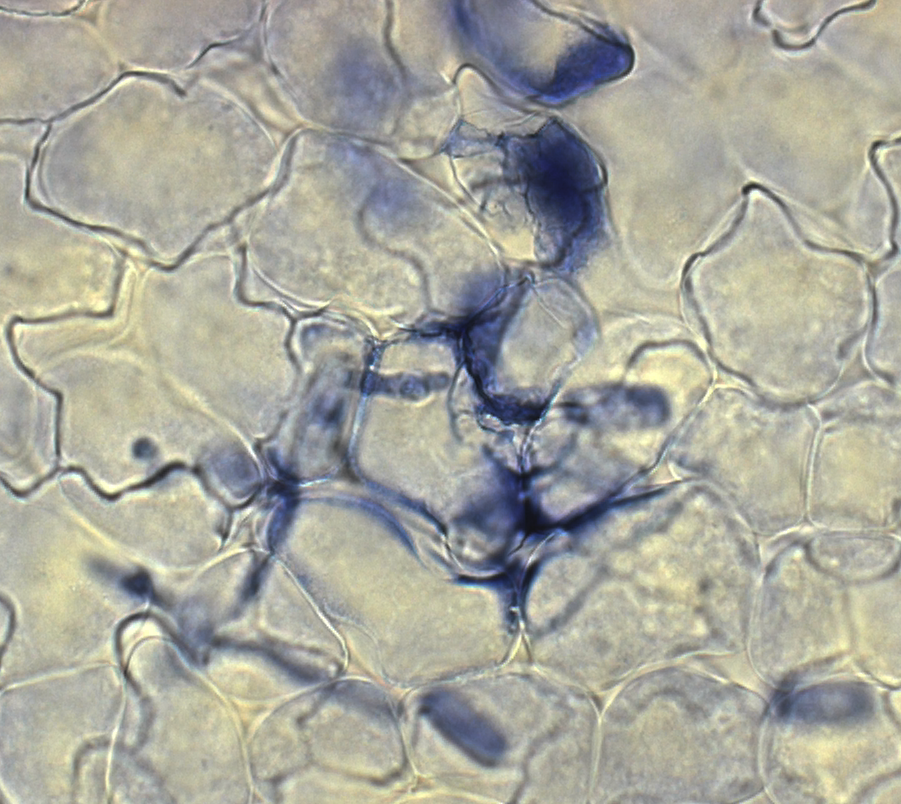


Kas-1 leaves were excised at 48 hpi and stained with trypan blue to detect plant cell death. Left: focus on epidermal cells interacting with the pathogen. Right: underlying mesophyll dead cells.
