# Supplementary figures and images for: Loss of compatibility might explain resistance of the Arabidopsis thaliana accession Te-0 to Golovinomyces cichoracearum
Source: BMC Plant Biol. 2012 Aug 11;12:143. doi: 10.1186/1471-2229-12-143 (PMC3546952; doi:10.1186/1471-2229-12-143)

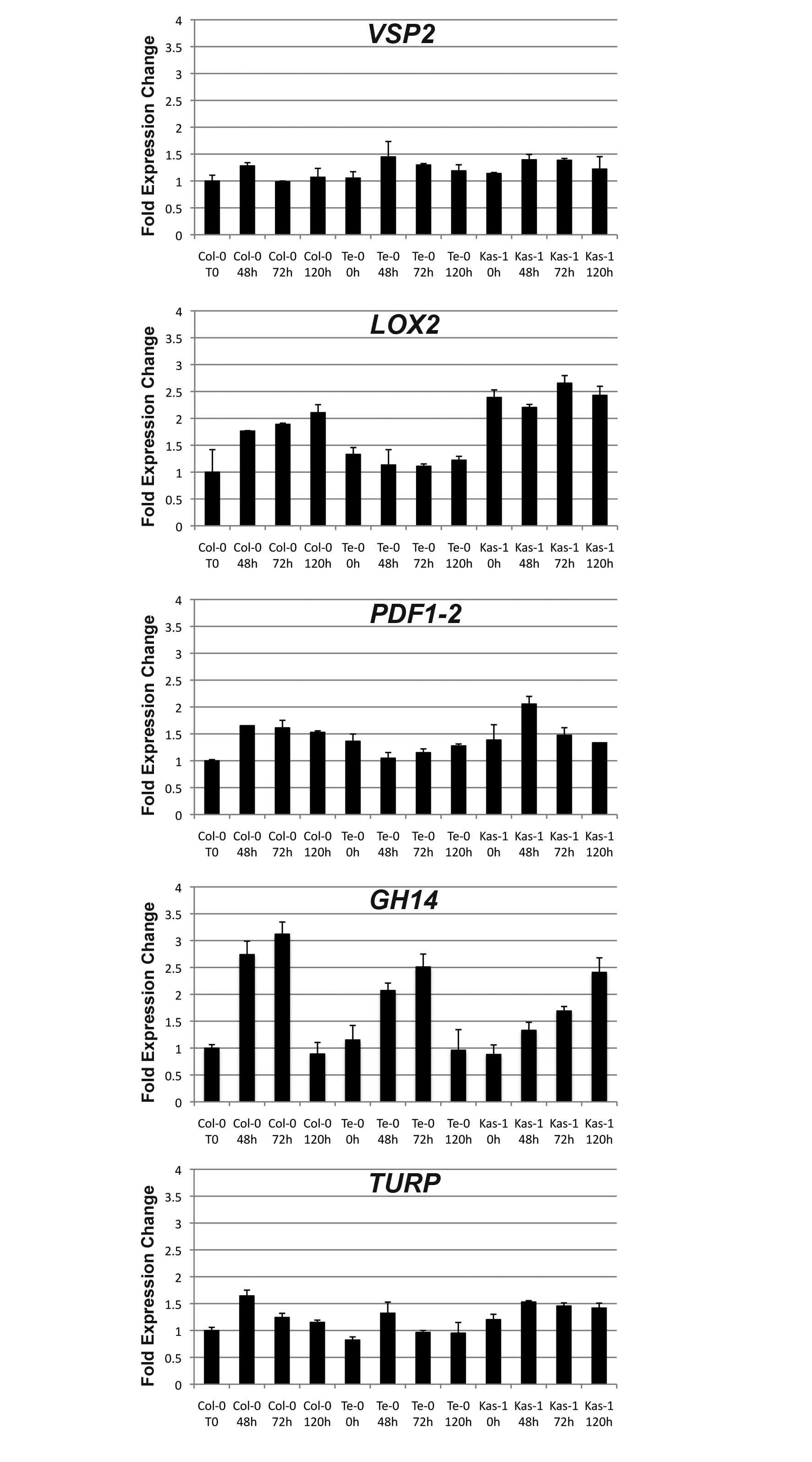

Supplement: Additional file 2 — Expression of defence genes on G. cichoracearum-infected tissues assessed by qRT-PCR. Values were obtained applying the ΔΔCt method using UBQ5 as housekeeping gene control. Bars represent average ± SD from three replicates. [file 1471-2229-12-143-S2.jpeg]

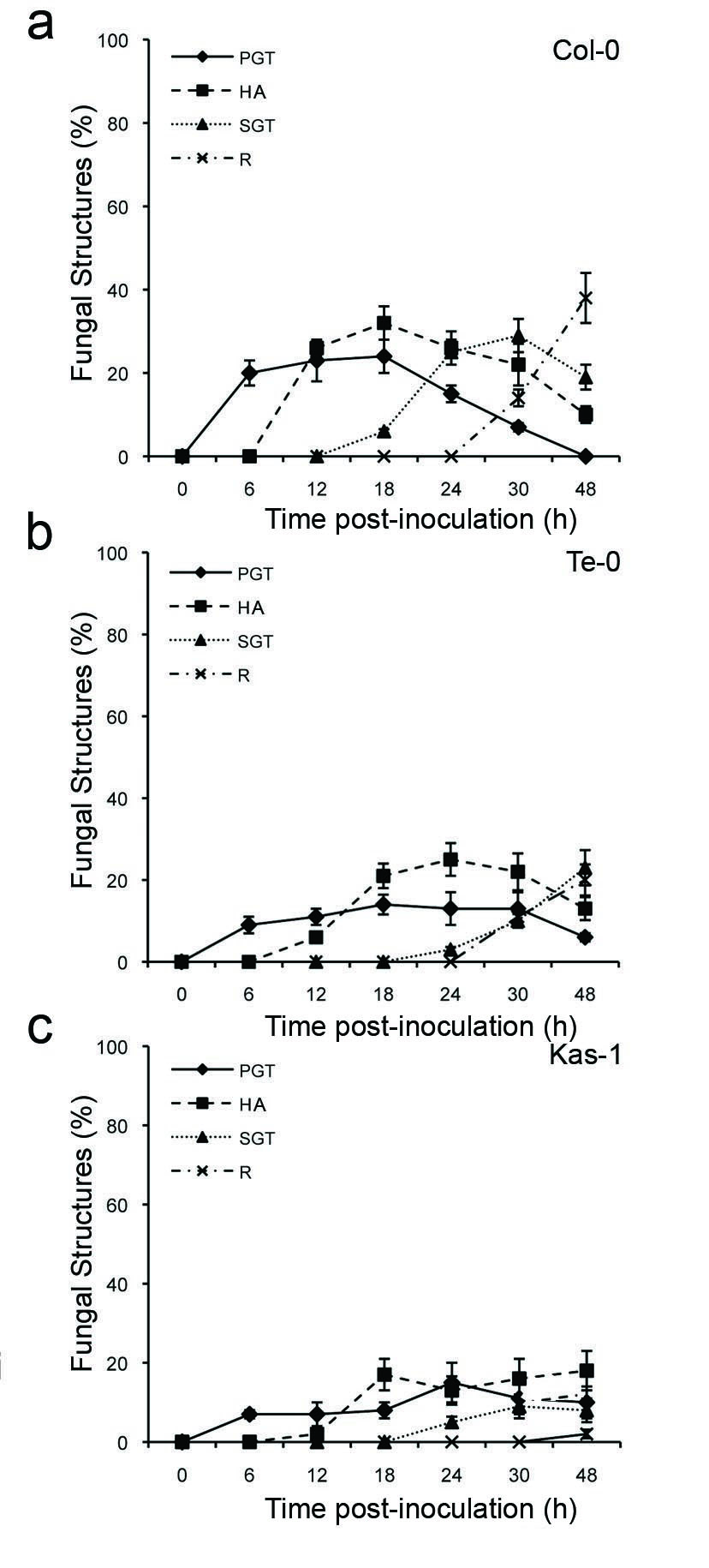

Supplement: Additional file 3 — Time course development of G. cichoracearum infective structures on Col-0, Te-0 and Kas-1 accessions. The abundance of different fungal structures is evaluated at the indicated time points. PGT: primary germ tube, HA: haustorium, SGT: secondary germ tube, R: ramified hyphae colony. Tissues were inoculated with the same conidial suspension. Values are referred as percentages of inoculated conidia. [file 1471-2229-12-143-S3.jpeg]

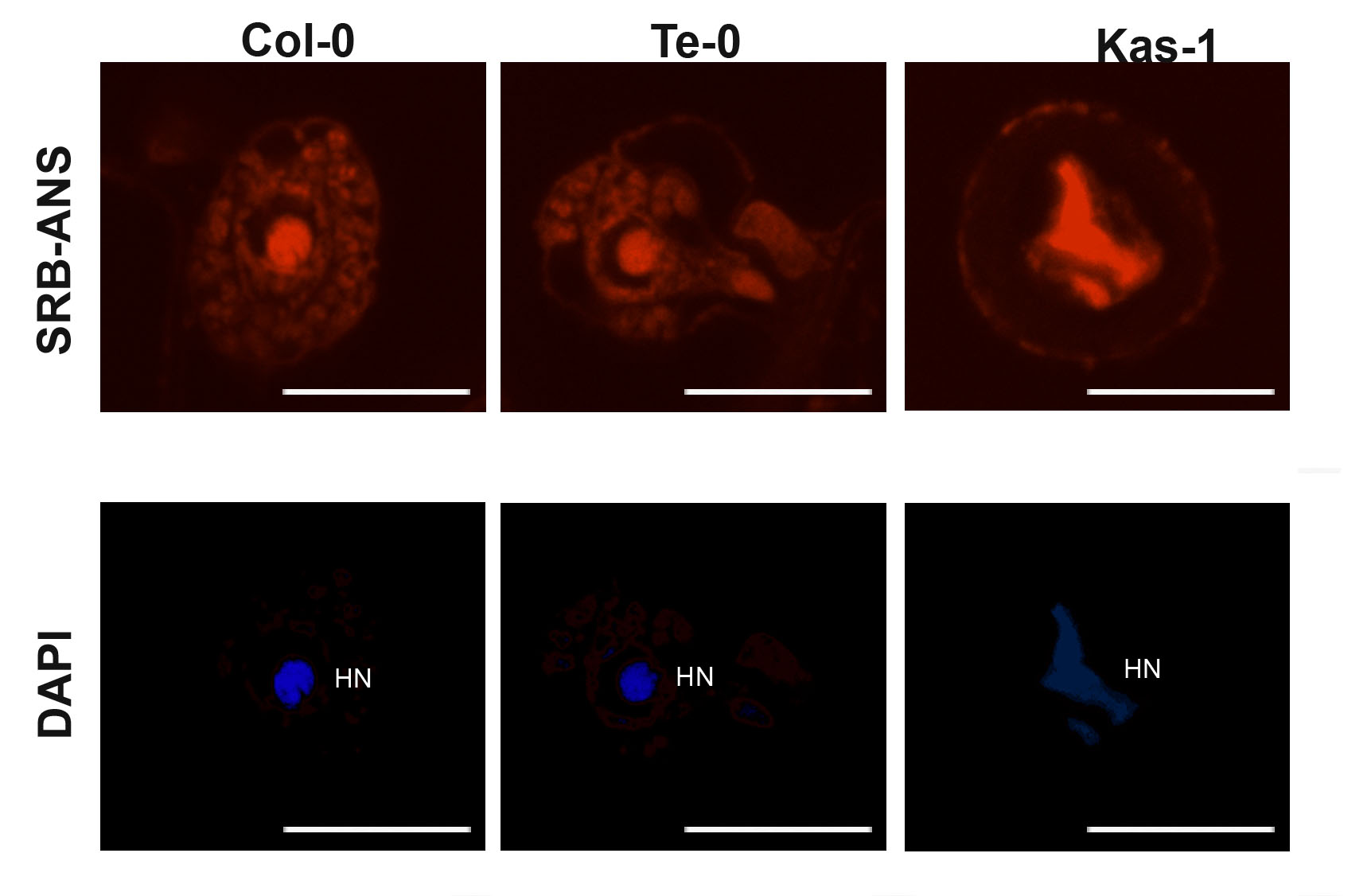

Supplement: Additional file 4 — DAPI staining of the G. cichoracearum haustoria developed in Col-0, Te-0 and Kas-1 plants. Haustoria were co-stained with SRB, ANS and DAPI. Z-stacks are shown for representative haustoria at 72 hpi. HN: haustorial nucleus. Bars: 10 μm. [file 1471-2229-12-143-S4.jpeg]

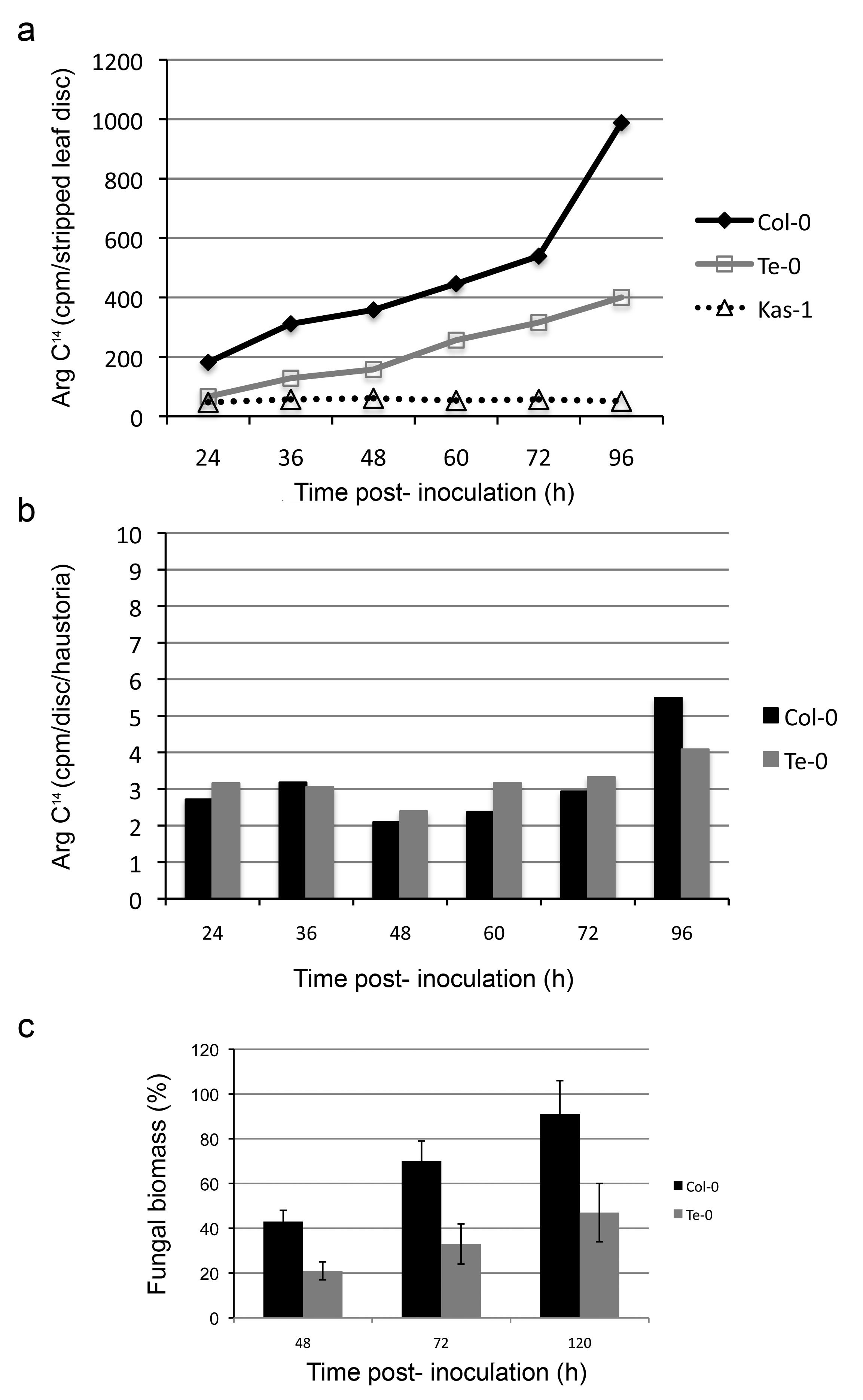

Supplement: Additional file 5 — Uptake of ArgC14 by G. cichoracearum haustoria installed in Te-0 cells. a) Uptake of ArgC14 by the fungal tissues differentiated on Col-0, Te-0 and Kas-1 leaves. The indicated values were obtained after subtracting residual radioactivity levels present on tapes stripped from uninfected leaves (see experimental procedures). b) ArgC14 levels detected in fungal tissue expressed as a ratio over the number of haustoria present on sibling leaf discs. c) Time-course estimation of the fungal biomass found on the surface of Col-0 and Te-0 leaves infected with G. cichoracearum. Fifteen leaves per plant were analyzed per time-point. Average percentage ± SD of leaf areas covered by fungal colonies is depicted. [file 1471-2229-12-143-S5.jpeg]

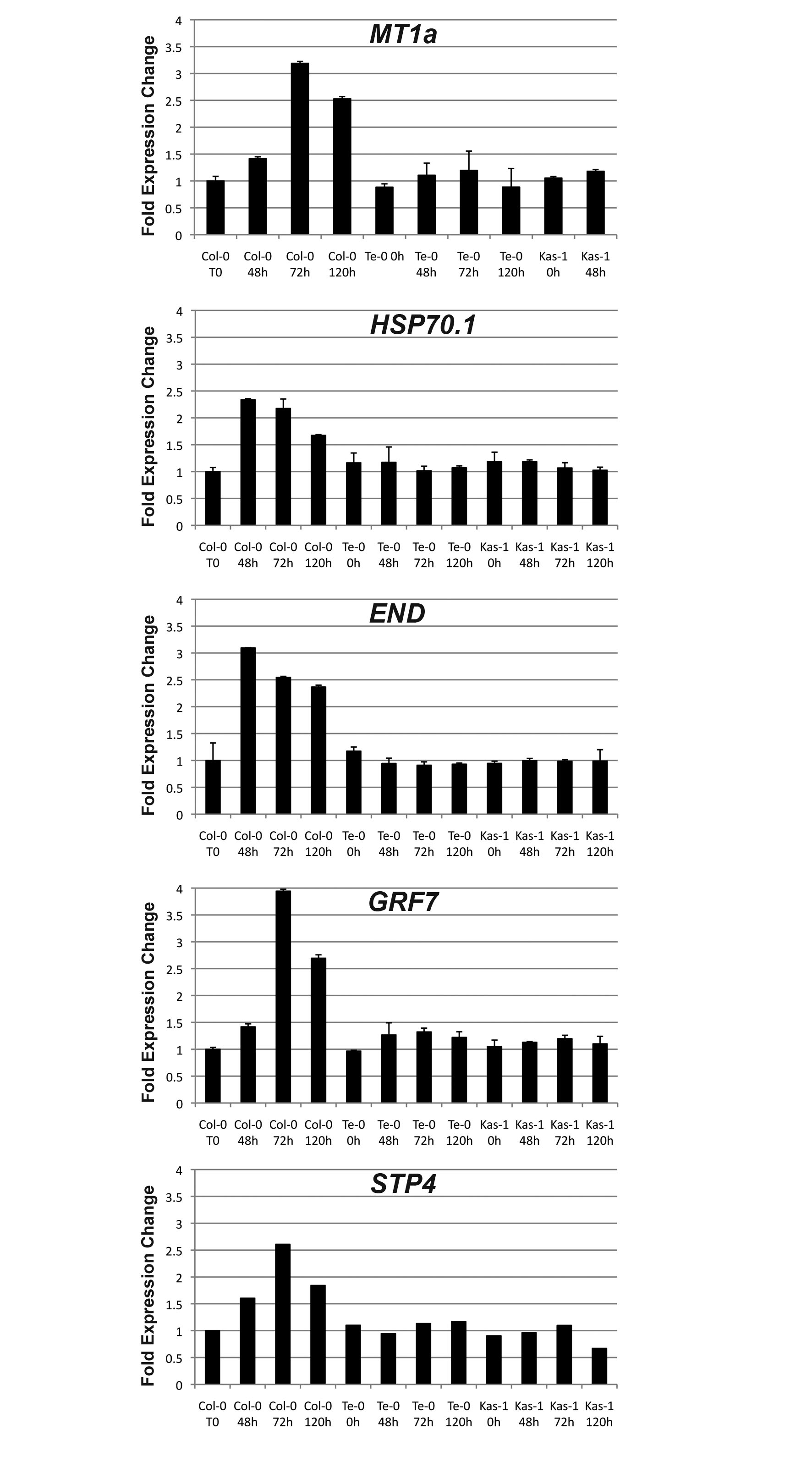

Supplement: Additional file 6 — Genes with differential expression in susceptible Col-0 and Te-0 resistant plants during the interaction with G. cichoracearum evaluated by q-RT-PCR. Fold changes in transcript expression levels were determined as described in Additional file 2. Bars represent average ± SD from three replicates, except for STP4, where q-PCR assay included two replicates. [file 1471-2229-12-143-S6.jpeg]

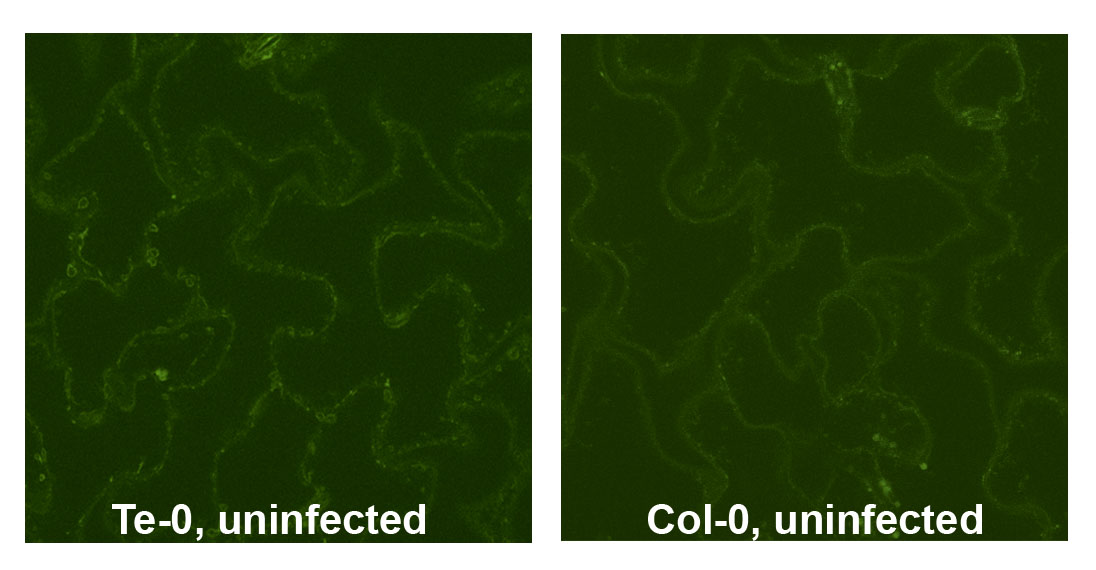

Supplement: Additional file 7 — Uninfected Te-0 plants do not accumulate callose. Untreated leaves from Te-0 plants (5-week-old, soil grown) were collected and stained with aniline blue to reveal callose by confocal microscopy using UV light. This assay was performed in parallel, under identical conditions to that shown in Figure 1b, where deposition of callose was detected in Kas-1 infected samples. Col-0 was included for comparison. [file 1471-2229-12-143-S7.jpeg]
