# Supplementary material for: Loss of compatibility might explain resistance of the Arabidopsis thaliana accession Te-0 to Golovinomyces cichoracearum
Source: BMC Plant Biol. 2012 Aug 11;12:143. doi: 10.1186/1471-2229-12-143 (PMC3546952; doi:10.1186/1471-2229-12-143)
Supplement: Additional file 8 — Primers and conditions used for semi-quantitative and real time/quantitative RT-PCR assays. (Ψ) Number of cycles used for sq-RT-PCR. (*) Different melting temperatures (Tm) were used in sq and q-RT-PCR. All q-RT-PCR were done with Tm = 60°C, except for PDF1-2. (^) UBQ5 and GapC were used as housekeeping control genes in q-RT-PCR and sq-RT-PCR, respectively. [file 1471-2229-12-143-S8.doc]

**Additional file 8. Primers and conditions used for semi-quantitative and real time/quantitative RT-PCR assays.**

| **Gene** | **AGI N°** | **Primers** | **Cycles**  **(Ψ)** | **Tm (°C)** | **Fragment size (bp)** | **Primer Efficiency (%)** |
| --- | --- | --- | --- | --- | --- | --- |
| *PDF1.2* | *At5g44420* | forward 5´-TAAGTTTGCTTCCATCATCACCC-3´  reverse 5´-GTGCTGGGAAGACATAGTTGCAT -3´ | 28 | 58 | 209 | 94 |
| *GH14* | *At4g17090* | forward 5´-TGCTTGGTGGGGATTGGTGGAG -3´  reverse 5´-CGCAATAACTCCTCCTATGTAGC -3´ | 26 | 60 | 371 | 100 |
| *TURP* | *At3g16640* | forward 5´-GGGAAGTAGAAGGAAAGTGGGT -3´  reverse 5´-ATGCATACCCTCCCCAACAAAGAA -3´ | 26 | 60 | 332 | 100 |
| *LOX2* | *At3g45140* | forward 5´-TACTTGCCTTCCCAAACACC-3´  reverse 5´- AGTGCCCTTGGCTGTAGAGA-3´ | 25 | 60 | 328 | 96 |
| *VSP2* | *At5g24770* | forward 5´-GAAGCCTAATGGTTCGAACTTG-3´  reverse 5´-CACGAGACTCTTCCTCACCTTT -3´ | 26 | 58/60* | 57 | 95 |
| *MT1a* | *At1g07600* | forward 5´-TAACTGTGGATGTGGCTCCTC -3´  reverse 5´- AGCTGCAGTTTGATCCACAGC -3´ | 27 | 60 | 111 | 110 |
| *HSP70.1* | *At5g02500* | forward 5´-CTCTGCTGAGGACAAGACCAC -3´ reverse 5´- TTCTCGTCTTGGATGGTGTTC -3´ | 27 | 60 | 211 | 100 |
| *END* | *At4g24190* | forward 5´-ACGATGTCAAAATCAGCAACC -3´ reverse 5´- CCACTCTCGATCAAAGCTGTC -3´ | 27 | 60 | 279 | 100 |
| *GRF7* | *At3g02520* | forward 5´-AGGAAGAAAGCAGAGGAAACG -3´  reverse 5´- AAGCAACCAGAGTGCTCTCAG -3´ | 27 | 60 | 249 | 100 |
| *STP4* | *At3g19930* | forward 5´-TTGGAGTCAAATTTGGAGTGG -3´ reverse 5´- GGACTTCTGTTGCATCTCAGC -3´ | 27 | 60 | 445 | 94 |
| *UBQ5* (^) | *At3g62250* | forward 5´-GTGGTGCTAAGAAGAGGAAGA -3´ reverse 5´- TCAAGCTTCAACTCCTTCTTT-3´ | -- | 60 | 251 | 102 |
| *GapC* (^) | *At3g04120* | forward 5´-CACTTGAAGGGTGGTGCCAAG-3´  reverse 5´-CCTGTTGTCGCCAACGAAGTC-3´ | 25 | 60 | 550 | ---- |

(Ψ) Number of cycles used for sq-RT-PCR.

(*) Different melting temperatures (Tm) were used in sq and q-RT-PCR. All q-RT-PCR were done with Tm = 60 °C, except for *PDF1-2*.

(^) *UBQ5* and *GapC* were used as housekeeping control genes in q-RT-PCR and sq-RT-PCR, respectively.
